# Effect of Motor Imagery on Serial Reaction Time Task Performance in Healthy Adults: A Randomized Crossover-Controlled Trial

**DOI:** 10.7759/cureus.82938

**Published:** 2025-04-24

**Authors:** Jaruwan Prasomsri, Katsuya Sakai, Yumi Ikeda

**Affiliations:** 1 Graduate School of Human Health Sciences, Department of Physical Therapy, Tokyo Metropolitan University, Tokyo, JPN; 2 Physical Therapy Department, Faculty of Medicine, Prince of Songkla University, Songkhla, THA; 3 Department of Physical Therapy, Faculty of Health Sciences, Tokyo Metropolitan University, Tokyo, JPN

**Keywords:** brain activity, functional near-infrared spectroscopy, motor imagery, physical practice, serial reaction time task

## Abstract

Introduction: This study used a serial reaction time task (SRTT) to explore motor learning, especially in complex sequential movements, and aimed to uncover the unique contributions of Motor imagery (MI) in optimising motor learning outcomes.

Methods: We aimed to explore the effect of MI or physical practice (PP) alone or a combination (CB) of both methods on SRTT in terms of premotor time, reaction time, accuracy rate, and brain activity immediately after training. All participants collected data on RT, the accuracy rate of response, and brain activity.

Results: Remarkable improvements in RT were observed post-training across all conditions, whereas the correctness rate remained unchanged. A marked reduction in dorsolateral prefrontal cortex (DLPFC) activity was noted in all conditions, with decreased activity in the premotor and supplementary motor cortices (PMC and SMA) in the MI and CB conditions. Notably, only the PP condition showed increased activity in the primary motor cortex (M1).

Conclusion: Our study underscores the significant enhancement resulting from CB conditions, showing similar improvements between the MI and PP methods. This suggests that both the combination and individual use of MI or PP can enhance physical performance, as evidenced by the improvements in reaction time and brain activity observed in our study.

## Introduction

Incorporating mental practice into training programs can enhance motor learning by involving learning procedures without motor execution [1]. Changes in motor performance from training result from neuromuscular adaptation during physical practice [2]. Motor imagery (MI), the mental reproduction of motor actions without muscle engagement, enhances motor learning [1-3]. Clinical studies demonstrate that MI, as a complementary treatment in rehabilitation, can improve physical performance beyond standard training, benefiting both neurological patients and athletes [1, 2, 4, 5]. MI is hypothesized to simulate internal models, utilizing predicted action outcomes to detect errors and facilitate learning. Despite its cognitive demands and being less effective than physical practice, MI enhances performance by anticipating errors through forward models. It improves perceptual-cognitive representations, influencing performance via perceptual anticipation, with learning effects varying by task and expertise [6, 7].

Further research is necessary to elucidate how MI, lacking sensory feedback, improves physical performance. Studies indicate that MI is more effective when combined with physical practice [4, 8]. Although physical practice remains the optimal method to enhance performance, combined training approaches yield benefits such as skill facilitation, reduced response times, and improved performance in precise tasks [4, 8]. These benefits are observed in athletic and rehabilitation settings, particularly enhancing upper limb function, gait speed, balance, and overall motor performance in stroke patients [1, 2, 4, 5].

Motor imagery has been widely studied in the context of sequence learning, particularly using serial reaction time tasks and similar paradigms. Research suggests that MI can facilitate sequence learning much like physical practice, though its effectiveness may vary depending on task demands and individual differences [1]. Studies have shown that MI alone can lead to the formation of sequence representations, supporting its role in implicit learning processes. One key finding in this area is the effector-independence of MI-based sequence learning. When individuals practice sequences through MI, they can transfer learned skills between effectors, such as from one hand to the other, suggesting that MI strengthens central motor representations rather than limb-specific execution patterns [1]. MI has also been shown to facilitate bimanual transfer, where training one hand improves performance in the other. These findings highlight MI’s potential as a tool for motor learning and rehabilitation.

Reaction time (RT) is a key performance metric reflecting sensorimotor coordination and alertness, influenced by central and peripheral nervous systems [9]. RT encompasses premotor and motor time. Premotor time is the interval from stimulus onset to EMG activity detection, representing central nervous system processes [10, 11]. Motor time is the duration between muscle activation and the finger-lift response. Thus, RT = PMT + MT [12]. Increasing task complexity extends premotor time, as indicated by EMG signals [10]. Researchers suggest MI affects premotor time by enhancing corticospinal excitability [10]. The serial reaction time task (SRTT) examines motor learning of complex, sequential movements. Participants respond quickly and accurately to serial stimuli, improving reaction times through practice and learning to anticipate stimuli [13]. The SRTT measures implicit learning, with participants often unaware of the underlying stimulus pattern. SRTT is valuable for studying implicit sequence learning [14], providing insights into motor learning via motor imagery, physical practice, or both [15]. Motor imagery is effective for skill acquisition, especially when combined with physical practice. Physical practice before motor imagery introduces sensorimotor information. Implicit sequence learning, measured independently from physical practice, shows the impact of motor imagery training [15].

Motor tasks are categorized as unimanual (one hand) or bimanual (both hands) [13]. Bimanual tasks are more complex, requiring higher coordination. A bimanual SRTT is often used to study learning in bimanual tasks, as many daily activities involve coordinated movements of both hands. This tool aims to understand the coordination and learning in bimanual activities, highlighting brain areas responsible for planning voluntary movements [13]. 

Ying (2012) found that brain areas involved in movement planning can be detected with activity differences across trials with varying RT following mental imagery (MI) or physical practice [16]. Participants practiced a sequence tapping task for seven sessions without prior sequence knowledge, allowing examination of cortical activity changes [16]. This underscores the impact of mental and physical practice on motor learning and performance.

Investigating RT and motor learning through choice RT and bimanual SRTT provides insights into motor coordination and the effects of practice on motor learning [1, 17]. Neuroimaging studies show that both physical practice and MI activate overlapping brain regions, including the cerebellum, which is responsible for error detection and correction [6]. This indicates that MI can independently update the motor program [15]. While performance improvements through repeated task practice are common, distinguishing whether such changes stem from motor learning or general physical exercise remains essential. Neural evidence, such as changes in brain activation or connectivity, is critical to confirm that improvements reflect central motor learning processes rather than peripheral adaptations [15]. Integrating neurophysiological measures with behavioral outcomes can therefore clarify the underlying mechanisms of task performance gains. 

MI training in healthy individuals leads to faster response times and increased cortical activity during training [1, 15]. Studying brain activity is crucial to understanding MI's direct impact. Neuroimaging shows that MI involves motor-learning processes that reorganize neural connections related to movement [2, 18]. Functional near-infrared spectroscopy (fNIRS) studies confirm the activation of primary, premotor, and supplementary motor areas during MI [19]. Brain regions involved in voluntary movement planning detect activity differences among trials with varying RTs following MI or physical training, suggesting their role in sequence learning [20]. Many daily activities require coordinated, continuous movements involving both hands. Dahm (2023) examined action imagery and execution practice on motor automatization using the SRTT, finding both methods reduced reaction times, though action imagery was less effective, indicating weaker mechanisms compared to execution practice [20]. This study introduced a bimanual SRTT to investigate learning during bimanual tasks.

Previous research has predominantly focused on the impact of MI on physical performance, with limited studies investigating its effect on learning [1, 15, 21]. Evidence of neural processing is also required to elucidate whether reduced movement time results from motor learning or physical exercise [1]. Limited exploration exists regarding MI in sequential movements for short reaction times [22]. 

This study aims to investigate the effectiveness of motor imagery (MI) in motor learning using the Serial Reaction Time Task (SRTT). The general objective is to evaluate whether MI, physical practice, or their combination can improve motor performance and elicit neural changes indicative of motor learning, rather than mere physical adaptation. The specific objectives are: (1) to examine the effects of MI on reaction time and accuracy in sequential motor tasks; (2) to assess changes in premotor time as an indicator of movement preparation; and (3) to explore post-training brain activity to determine neural evidence supporting the use of MI in motor learning. 

We hypothesize that MI, physical practice (PP), and combined (CB) conditions will all result in reduced reaction time and brain activity post-training. MI is expected to lead to the greatest reduction in premotor time, while PP is anticipated to most significantly improve reaction time and accuracy. This is based on the assumption that MI primarily enhances planning time, whereas PP improves overall RT by reinforcing motor execution and response accuracy [21]. Participants engaging in a combination of MI and PP are expected to demonstrate greater improvements in reaction time and accuracy than those utilizing either method alone [20]. We also hypothesize distinct neural processing patterns for MI compared to PP, aligning with changes in premotor time and accuracy, indicating that CB induces unique motor learning effects.

## Materials and methods

Participants

The study recruited participants from among young adults between the ages of 18-36 years [23]. Sample size calculation, performed using G*Power 3.1 software (Heinrich-Heine-Universität Düsseldorf, Düsseldorf, Germany), indicated that a total of 42 participants were required to achieve a moderate effect size of 0.25 with a power of 0.80. This sample was to be divided equally across three conditions, with 14 participants in each condition. However, given the crossover study design, which allows each participant to experience all conditions, only 14 participants were ultimately needed. This design minimizes the need for separate groups for each condition, reducing the total number of participants required while still fulfilling the study's objectives. Participants are randomly assigned to different sequences of interventions, ensuring that each participant receives all interventions in varying orders. A seven-day washout period is implemented between interventions to minimize carryover effects [24]. Each participant serves as their own control by receiving all interventions, which allows for within-subject comparisons. 

Prior to being randomly assigned to specific training conditions, the participants underwent screening based on pre-established study criteria. The inclusion criteria required participants to be right-handed, as confirmed by the Edinburgh Handedness Inventory questionnaire [25]. They were also required to achieve a score above 25 on the Kinesthetic and Visual Imagery Questionnaire (KVIQ-20) to evaluate their MI, as well as normal hearing and verbal abilities. The KVIQ evaluates the vividness and clarity of kinesthetic (movement-based) and visual (image-based) motor imagery. It consists of 25 items, where participants rate the vividness of their imagery for tasks, such as lifting an object or walking, on a scale from 1 to 5, with higher ratings indicating clearer and more vivid imagery [18]. Conversely, participants who met the exclusion criteria, such as having neurological or psychiatric disorders, upper limb musculoskeletal issues, or prior exposure to sequence learning studies or externally stimulated experiments of the cerebral cortex (such as transcranial magnetic stimulation or transcranial direct current stimulation), and those who presented with colour blindness were not eligible to participate.

Procedures

**Figure 1:**
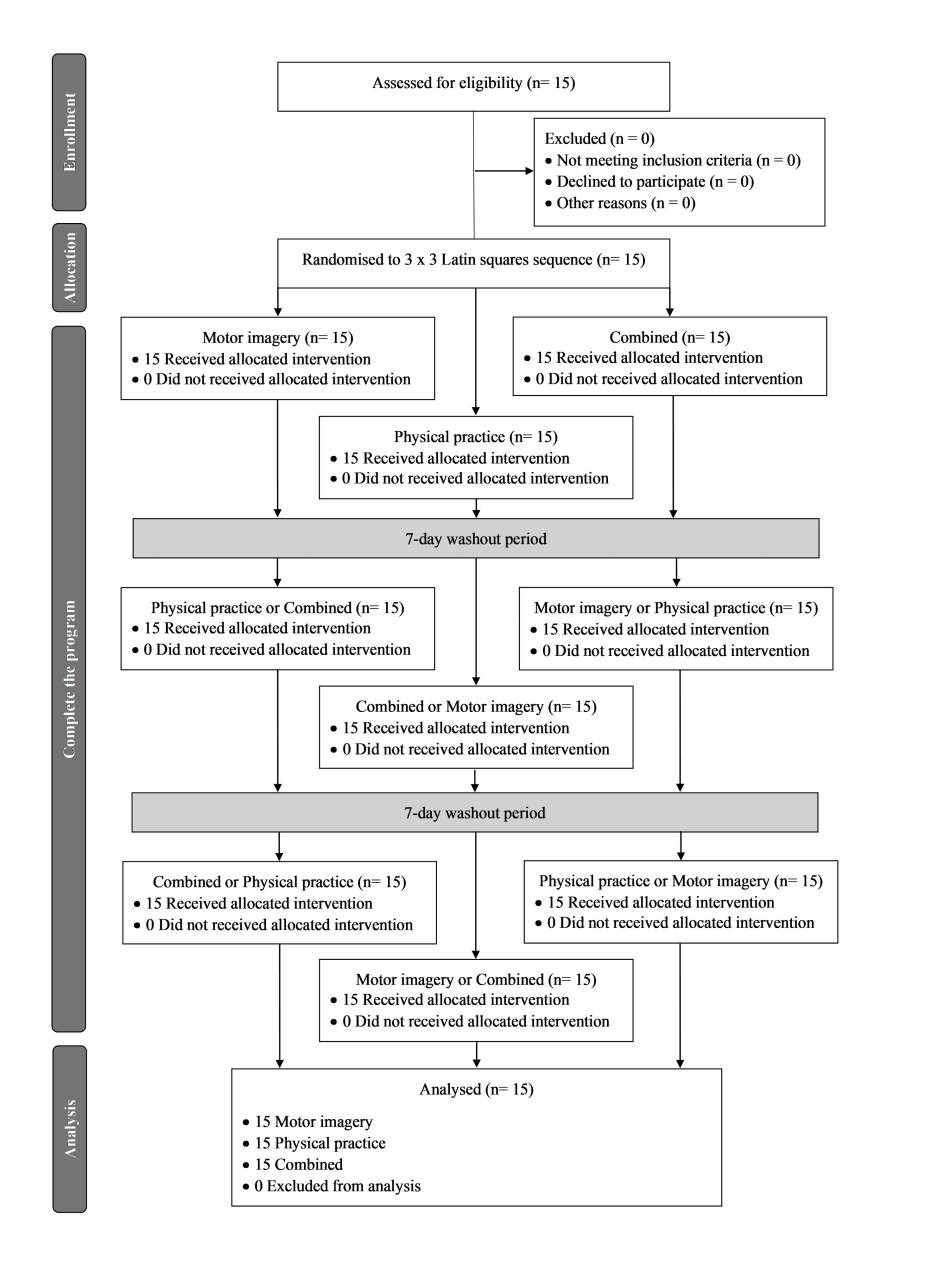
CONSORT flow diagram

Informed consent was obtained from all participants before enrolment in the study. The experiment was approved by the local ethics committee of Tokyo Metropolitan University in Tokyo, Japan, in accordance with the principles of the Declaration of Helsinki. This study (registration number: 22070) was approved on 17 March 2023. Additionally, this study has been registered with the Thai Clinical Trials Registry (TCTR) under registration number TCTR20230824008 and the University Hospital Medical Information Network Clinical Trials Registry (UMIN-CTR) under registration number UMIN000053083. The CONSORT diagram for this crossover study outlines the participant flow and study procedures. Initially, 15 participants were enrolled and met the eligibility criteria. Following the screening process, none of these participants were excluded, resulting in all 15 being randomized into the study. This study employed a single-blind, simple random sampling method using a lottery system. Each participant randomly selected a number themselves, without knowing the corresponding training condition. During the first phase of the crossover design, each participant was assigned to one of three conditions: Condition MI, Condition PP, or Condition CB. After a washout period designed to mitigate carryover effects, participants transitioned to an alternative condition for a duration of seven days. Upon completion of the intervention, data collection for the primary outcomes was conducted. The final analysis included data from all 15 participants, reflecting the complete participant flow through the study. A CONSORT flowchart of the study is presented in Figure 1.

Testing protocol

**Figure 2:**
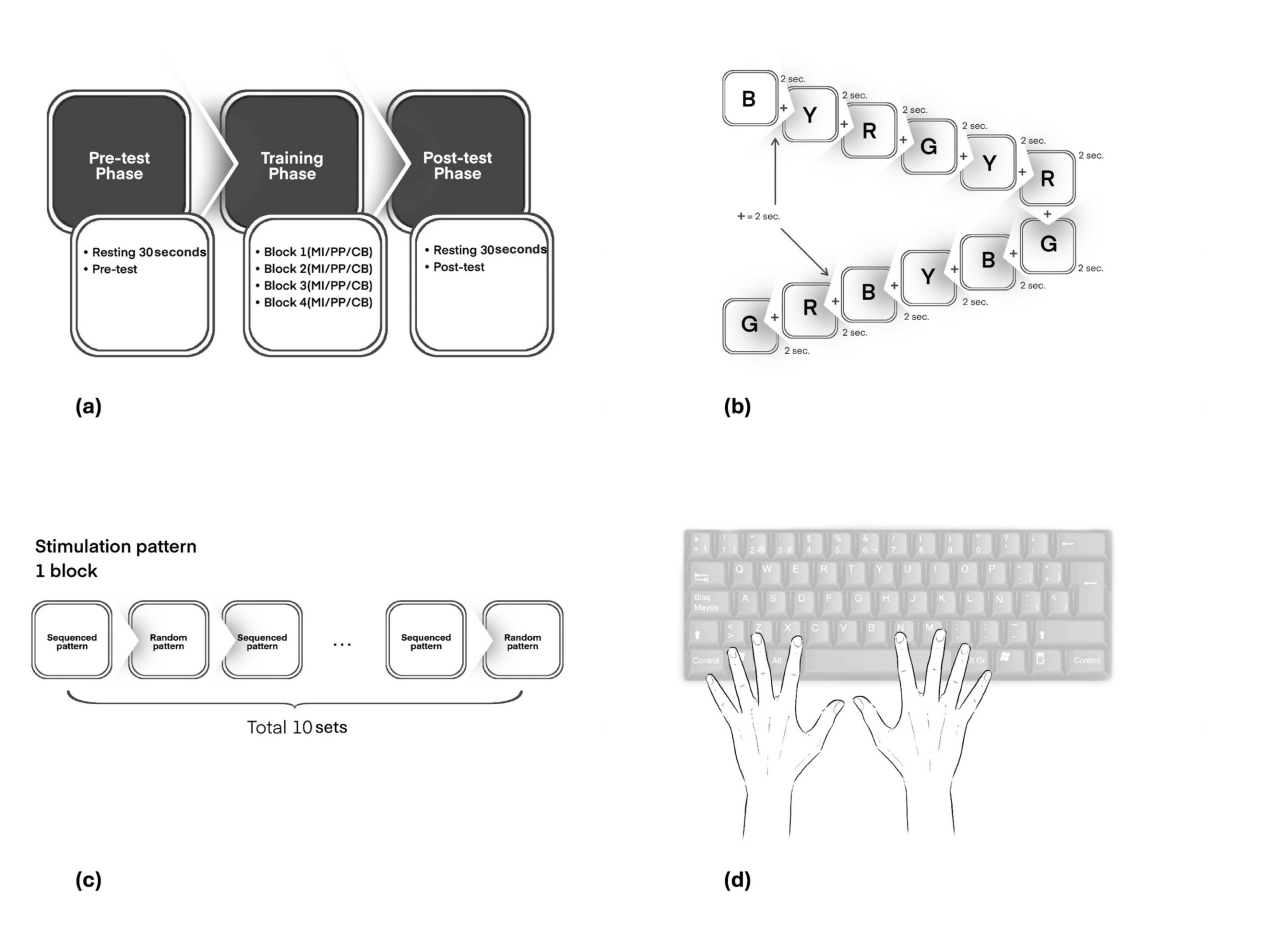
Testing protocol (a) Flow of the study. The diagram illustrates the overall progression of the study, detailing each phase from pre-test to post-test. (b) Example of the stimulation pattern sequence in one set. This panel shows a representative sequence of the stimulation patterns used in a single set. (c) Sequences alternating between sequenced and random patterns in 1 block. (d) Finger placement on the keyboard for stimulus response. Participants were instructed on the correct positioning of their fingers on the keyboard they were to use when responding to the stimuli during the study.

Each participant provided data on RT and brain activity while completing a single block of the SRTT. The average of all the responses was used as a representative measure of the data collected before and after the training sessions (Figure 2).

Training protocol

The training conditions included MI, physical training (PP), and combined MI and PP (CB). To guarantee accurate assessment of each condition without influence from prior conditions, this study utilised a randomised 3 × 3 crossover design (Table 1) with a seven-day break period between sessions. In each training session, the participants completed four training blocks, with a short pause after the initial two blocks. There was a five-minute rest period before continuing with the final two training blocks. Motor Imagery condition; participants were directed to mentally visualise their response to an SRTT displayed on a computer screen from a first-person or kinaesthetic perspective without making any physical movements. The participants placed their hands on the keyboard in the same manner as in the Physical Practice (PP) condition. In the PP condition, participants were instructed to physically react to an SRTT. For the Combined (CB) condition, participants were directed to imagine completing the task for three blocks and then engaging in physical responses for the remaining block.

**Table 1:** The 3 × 3 randomized crossover design of the study Motor imagery = A, Physical practice = B, Combination = C

Participants															
Group	1	2	3	4	5	6	7	8	9	10	11	12	13	14	15
	A	B	C	A	B	C	A	B	C	A	B	C	C	B	A
	B	C	A	C	A	B	B	C	A	C	A	B	A	C	B
	C	A	B	B	C	A	C	A	B	B	C	A	B	A	C

The participants were instructed to refrain from verbalising or mentally imagining any physical movement and were instead encouraged to focus on visualising themselves performing the task from a first-person perspective. During the remaining intervals, they were advised to engage in unrelated thinking. Additionally, the participants were told to imagine themselves performing the task throughout the entire imagery period and avoid any physical movement until the period was concluded.

Serial reaction time task

The RT experiment utilised the SuperLab software version 6.0 (Cedrus Corporation, San Pedro, CA). The participants were seated in a comfortable chair and maintaining approximately a 90-degree flexion of both elbows in front of the desk. Their objective was to respond promptly and accurately to the visual stimuli displayed on a computer screen by pressing the corresponding keys on the keyboard.

The task used in the experiment involved showing a series of four-colour pictures (red, green, yellow, and black) one after the other in the centre of the screen. Each round consisted of 12 pictures, repeated ten times to make up one set. This study included four training sets. Participants had to press specific keys for each colour: "Z" for red, "X" for yellow, "N" for green, and "M" for black. The keys "Z" and "X" were operated by the middle and index fingers of the left hand, while the keys "M" and "N" were operated by the middle and index fingers of the right hand. The goal was to respond quickly and accurately, with each picture displayed for 2 s, followed by a 2-second break represented by a plus sign (“+”) before the next picture appeared. The picture was displayed for 2 seconds, regardless of whether the participant responded or not.

The sequences alternated between testing and training phases, incorporating both sequenced and random patterns. The training and testing blocks were designed with the same pattern. The stimulation pattern in each block consisted of five sequenced patterns and five random patterns. Each training and testing block began with sequenced patterns, followed by random patterns, alternating in this manner until a total of 10 sequences were completed. The testing phase consisted of one block, while the training phase comprised four blocks. Data were collected before and after the experiment, following a demonstration and practice round.

In the MI condition, the order of colours was ‘black-yellow-red-green-yellow-red-green-black-yellow-black-red-green’. The PP condition arranged the colours as ‘yellow-black-red-green-black-yellow-red-black-green-red-yellow-green’. The CB condition employed the sequence ‘red-black-red-green-yellow-green-red-yellow-green-black-yellow-black’. Each condition had an equal colour distribution to avoid bias. To ensure that the training session memory did not interfere, each condition had a unique colour sequence in accordance with the crossover study design.

The duration of each block of the SRTT was approximately 8 min, and the total training session lasted 40 min, including breaks. The participants were advised to either close their eyes or focus on a blank screen during breaks. Each pre- and post-testing session lasted approximately 15 minutes. After the training session, a 5-min break was provided before conducting the post-test session. However, if participants required additional time for a break, they were allowed to rest until they felt ready to proceed with the post-test. Consequently, the total time required for testing and training under each condition was approximately 1 h. Following completion of each experimental condition, the participants were tasked with recalling the stimulus sequences as a measure of implicit memory.

Electromyography

The preparation involved attaching surface electrodes to both arms to capture data from the wrist extensor muscles, specifically targeting the Extensor Digitorum Communis. During testing and MI training, EMG signals are captured from the wrist extensor muscles, which are then processed by amplifying and filtering the captured EMG and accelerometer signals, sampled at 2000 Hz within a band-pass range of 5 Hz to 1 kHz; subsequently, utilising the DELSYS® EMG Signal Analysis program, the EMG analysis, the EMG signals were rectified and filtered using a low-pass filter with a cutoff at 10 Hz. This filter was chosen based on previous research optimizing noise reduction in rectified EMG signals [26]. Muscle activity during the 30-s resting phase preceding the pre- and post-tests was calculated. The threshold indicating muscle activity was defined as the average rest plus twice the standard deviation [15]. The aim of integrating EMG in this study was to detect the exact moment at which the target muscle began to contract. As a result, the commencement of the motor time for the reaction time task was established as the juncture at which the EMG signal surpassed the mean resting level by a factor of two times the standard deviation. If more than 15% of the comparisons exceeded the threshold, participants were excluded from subsequent analyses [15].

Functional near-infrared spectroscopy

Brain activity was evaluated using fNIRS, which measures brain activity by observing regional tissue oxygenation. During data collection, participants wore a head cap equipped with optodes that covered regions of interest, including the prefrontal cortex, supplementary motor area, primary motor cortex, and premotor area. This setup allowed fNIRS to monitor brain activity in these areas. This approach utilised during the 30-s resting period before the pre and post-tests, as well as throughout each testing and training block.

The fNIRS (Shimadzu Corp., Kyoto, Japan) system, equipped with a cap containing 30 optodes (light sources and detectors), was utilised, and the sensors were evenly distributed across both hemispheres following the International 10-20 system and accurately aligned with Cz. Oxyhaemoglobin (oxy-Hb) and deoxyhaemoglobin (deoxy-Hb) levels were selected for analysis, recorded at a sampling rate of 10 Hz, and processed using a bandpass filter with a low cutoff frequency of 0.01 Hz and a high cutoff frequency of 0.1 Hz [27]. Subsequently, a correction process was applied to set the data position at the baseline level at 3 seconds, aligning with the initial stimulus during the reaction time test. This adjustment aimed to eliminate background noise. Concentrations of oxy-Hb and deoxy-Hb were determined by averaging values during the resting phases before the pre-test, the pre-test phase, the resting phase before the post-test, and the post-test phase. For the pre-test data, the values were obtained by finding the difference between the pre-test phase and the resting phase before the pre-test. For the post-test data, the values were determined by subtracting the post-test phase values from the resting phase before the post-test. Brief relaxation periods lasting 30 s each were conducted before both the pre- and post-tests.

**Figure 3:**
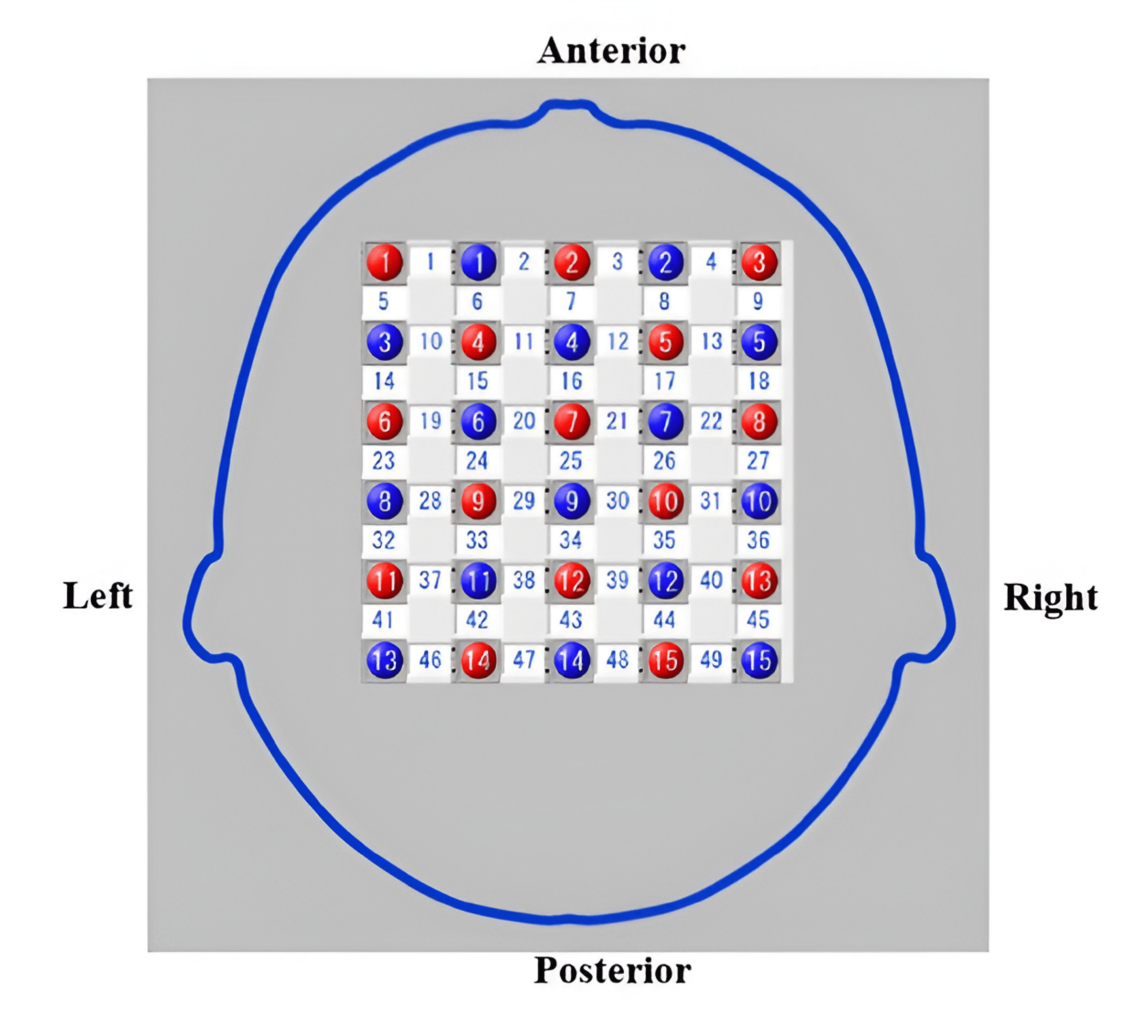
Channel of locations using functional near-infrared spectroscopy

The source and detector optodes were positioned 3 cm apart and digitised using a three-dimensional magnetic space digitiser (FASTRAK; Polhemus, Colchester, VT, USA), resulting in 49 channels of 5 × 6 (Figure 3). The modified Beer-Lambert-Law, implemented via the NIRS-SPM toolbox in MATLAB, was utilised to analyse oxy-Hb changes and identify activated regions.

Upon identifying the brain's location, the brain areas from the FASTRAK 3D system and Matlab® were employed for analysis. The channel exhibiting the greatest overlap in fNIRS was selected to represent each region of interest. However, if the channel with the highest value was omitted because of a calibration failure at the beginning of the test, the subsequent channel with the next highest value was selected for analysis.

Behavioural parameters

**Figure 4:**
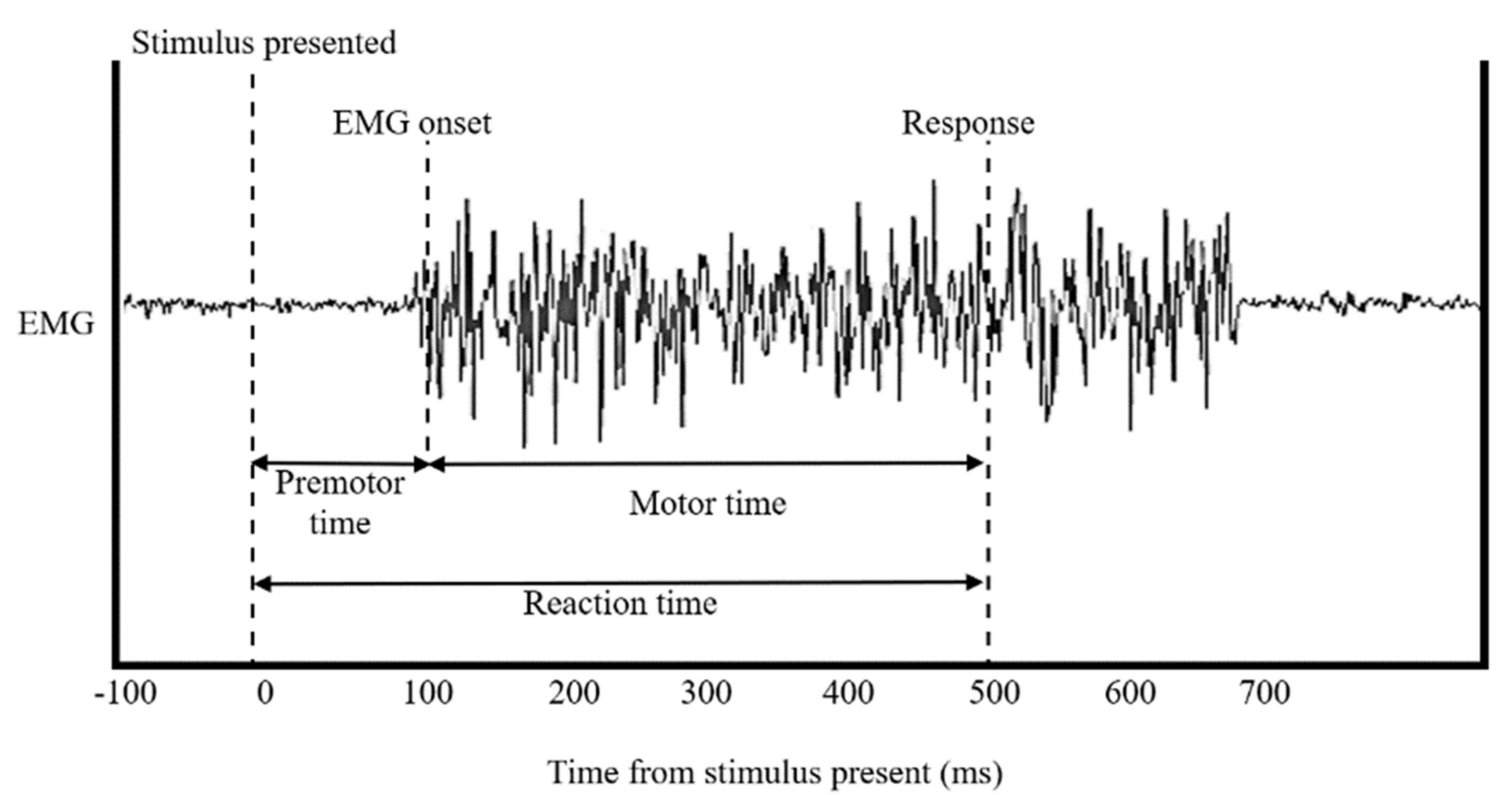
Components of RT

Reaction time in this study is defined as the interval between the presentation of a colour picture on the computer screen and the participant pressing a key on the keyboard. In this study, SuperLabs' reaction time calculations were analysed, with only the correct responses selected for analysis. The premotor time is the time from stimulus onset to the initiation of motor response preparation, reflecting cognitive processes involved in movement planning. The premotor time is calculated by detecting the onset of muscle activity or the start of movement after the stimulus (Figure 4). Reaction time and premotor time that were either above or below two standard deviations from the mean in each block were excluded from further analysis.

Brain activity parameters

The concentrations of oxy-Hb and deoxy-Hb were calculated by averaging each region during the resting phase before the pre-test, the pre-test phase, the resting phase before the post-test, and the post-test phase. The pre-test data were represented by the differences between the pre-test and resting phases before the pre-test phase. Similarly, post-test data were obtained by subtracting the post-test value from the resting value before the post-test. The resting phases, each lasting 30 s, were conducted before both the pre- and post-tests as brief relaxation periods.

Statistical analysis

Information regarding baseline characteristics such as sex, age, weight, and height, as well as measurements of RT and brain activity assessed via fNIRS, were collected before and after the training sessions. The statistical analysis in this study used non-parametric methods. The Shapiro-Wilk test showed that all variables had a non-normal distribution during the normality test. The assessment of the influence of the training conditions involved the use of a Wilcoxon Signed-Rank test and a Kruskal-Wallis test, considering both conditions and test sessions as variables. Following this analysis, multiple tests were adjusted using the false discovery rate (FDR), and statistical significance was defined as a p-value below 0.05.

## Results

During the training sessions, participants refrained from making any movement until the conclusion of each training block. Assessments revealed no activity in the extensor digitorum communis muscles, with thresholds below 15% of the baseline comparison during the MI task. Only a small subset of participants, specifically three individuals, claimed to be able to recall sequences, and the accuracy of their recall was less than 25% of that of the training sequence, indicating implicit learning [21]. Most participants reported an inability to recall sequences under any training condition. No dropouts or adverse effects were reported during or after the training sessions.

Participant characteristics

Fifteen volunteers were assessed for eligibility, with none being excluded. Consequently, all 15 participants completed the training under all three conditions. Therefore, data from these 15 healthy young adults, who met the study requirements and successfully completed the training, were used for the final analysis. The baseline characteristics are shown in Table 2. The Shapiro-Wilk test showed that all variables had a non-normal distribution during the normality test. 

**Table 2:** Baseline characteristics BMI, body mass index; KVIQ: Kinesthetic and Visual Imagery Questionnaire

Factors (N = 15)	Median (Q1, Q3)
Sex Male = 4, 26.7% Female = 11, 73.3%	
Age (year)	28 (26, 30)
BMI	18.26 (16.96, 19.09)
Education years (year)	18 (17, 19)
KVIQ visual	43 (38, 46)
KVIQ kinesthetic	43 (29, 45)

Behavioural parameters: reaction time, premotor time, and accuracy rate

**Figure 5:**
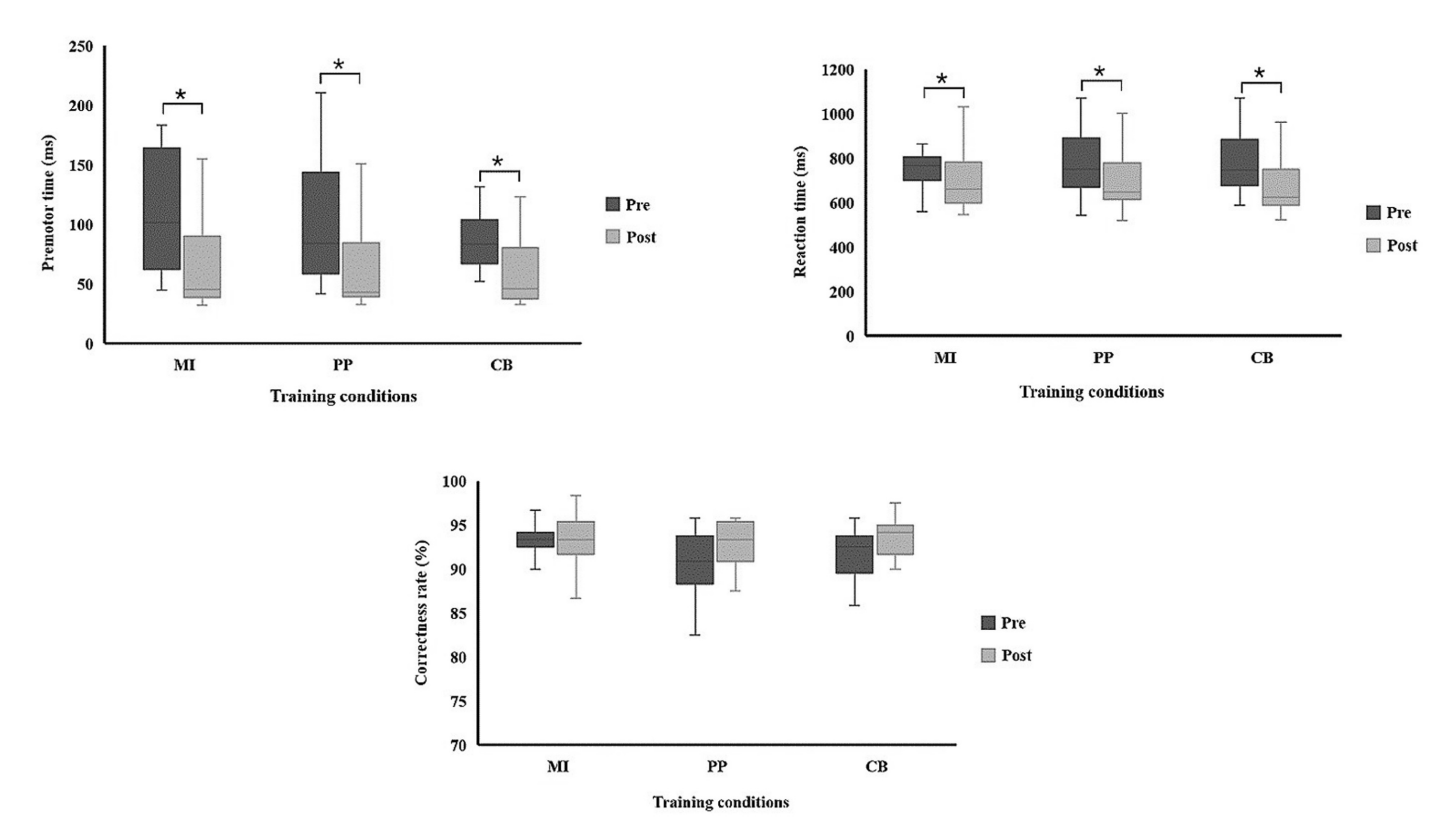
A comparison of the physical parameters within each condition MI, motor imagery; PP, physical practice; CB, combination between motor imagery and physical practice: ms, milliseconds. * False discovery rate-corrected p-values of < 0.05

In this study, behavioural parameters were calculated from both sequenced and random patterns during testing blocks. There were no significant baseline differences among the three conditions across all measured parameters. The premotor time, reaction time (RT), and correctness rate for the three conditions are summarized in Tables 3-4, showing median values and interquartile ranges for within-condition and between-condition comparisons. Post-training analysis revealed significant reductions in both premotor time and RT within each condition, with an FDR-corrected p-value < 0.05 (Figure 5, top left and right panels, respectively). Specifically, the PP condition exhibited the shortest premotor time across all (p-value <0.001), while the CB condition showed a significant reduction in RT compared to the other conditions (p-value <0.001). These improvements in premotor time and RT were not accompanied by any significant improvements in correctness rate under any condition (p = 0.250). The shortest premotor time was observed in the PP condition when compared to both the MI and CB conditions. Additionally, the CB condition showed the shortest RT compared to both PP and MI. Specifically, RT was shorter in the PP condition than in the MI condition, as indicated by the single comparison of MI vs. PP. The detailed results are illustrated in Figure 5, with statistical significance indicated by p < 0.05 (FDR-corrected).

**Table 3:** Reaction time variables for all conditions RT, reaction time; MI, motor imagery; PP, physical practice; CB, combination of motor imagery and physical practice; ms, milliseconds. P-values: False discovery rate corrected.

Condition	Variable	Pre	Post	P-value
		Median (Q1, Q3)	Median (Q1, Q3)	
MI	Premotor (ms)	101.5 (62 ,166.9)	45.3 (38.5,130.8)	< 0.05
	RT (ms)	765.4 (697.5,809.7)	659.5 (594.5,792.5)	< 0.05
	Correctness rate (%)	93.3 (92.5,94.2)	93.3 (91.7,95.8)	> 0.05
PP	Premotor (ms)	84.2 (58,164.3)	42.7 (38.9,88.3)	< 0.05
	RT (ms)	749.8 (667.9,896.5)	646.1 (603.9,832.8)	< 0.05
	Correctness rate (%)	90.8 (87.5,94.2)	93.3 (90.8,95.8)	> 0.05
CB	Premotor (ms)	83 (65.3,103.9)	46.3 (37.5,94.6)	< 0.05
	RT (ms)	746 (668.3,889.8)	626.1 (586.6,787.3)	< 0.05
	Correctness rate (%)	92.5 (89.2,94.2)	94.2 (91.7,95)	> 0.05

**Table 4:** Comparison of the results of reaction time variables between conditions RT, reaction time; MI, motor imagery; PP, physical practice; CB, combination of motor imagery and physical practice; ms, milliseconds. P-values: False discovery rate corrected.

Variable	MI	PP	CB	P-value
	Median (Q1, Q3)	Median (Q1, Q3)	Median (Q1, Q3)	
Premotor (ms)	45.5 (38.5,130.8)	42.7 (38.9,88.3)	46.3 (37.5,94.6)	< 0.05
RT (ms)	659.5 (594.5,792.5)	646.1 (603.9,832.8)	626.1 (586.6,787.3)	< 0.05
Correctness rate (%)	93.3 (91.7,95.8)	93.3 (90.8,95.8)	94.2 (91.7,95)	> 0.05

Brain activity: fNIRS results

These 23 channels correspond to three distinct regions of interest (ROI): the dorsolateral prefrontal cortex (DLPFC), premotor and supplementary motor cortex (PMC and SMA), and primary motor cortex (M1). Specifically, the DLPFC comprised channels 15-17, 19-23, and 27; the PMC and SMA encompassed channels 28-33, 35, 36, 38, and 39; and the M1 comprised channels 37, 40, and 42-44.

The selection of channels was based on previous fNIRS studies on motor learning. MI is known to primarily engage the prefrontal and premotor areas, whereas PP elicits greater activation in the primary motor cortex (M1). We recognize that a more explicit theoretical justification should have been provided and will address this in the manuscript revision. During the analysis, we used the same ROIs across all conditions. The channels presented in the table represent only those within the ROI that showed significant activation in each condition.

The oxy-Hb and deoxy-Hb data obtained from fNIRS for the three conditions outlined in Tables 5-7 were analysed for within-condition comparisons and between conditions, respectively, and are presented as the median and 1st and 3rd quartiles. Following training, a statistically significant difference was observed within each condition, with an FDR-corrected p-value < 0.05. All conditions exhibited a reduction in activity within the DLPFC. Specifically, two conditions (MI and CB) displayed decreased activity within the PMC and SMA across all conditions. Notably, only the PP condition demonstrated enhanced activity in the M1.

**Table 5:** Brain activity (oxy-HB) under all conditions Ch channels; Lt, left; Rt, right; Oxy-HB, oxy-haemoglobin; ROI, regions of interest; DLPFC, dorsolateral prefrontal cortex; PMC, premotor cortex; M1, primary motor area; SMA, supplementary motor cortex; MI, motor imagery; PP, physical practice; CB, combination between motor imagery and physical practice. P-values: False discovery rate corrected.

Condition	Channels	ROI	Pre	Post	P-value
			Median (Q1, Q3)	Median (Q1, Q3)	
MI	22	Rt DLPFC	0.013 (-0.001,0.048)	-0.008 (-0.028,0.006)	< 0.05
	32	Lt PMC and SMA	0.010 (0.001,0.029)	-0.006 (-0.016,0.010)	< 0.05
	39	Rt PMC and SMA	0.013 (-0.002,0.031)	-0.003 (-0.017,0.011)	< 0.05
PP	15	Lt DLPFC	0.011 (0.003,0.035)	-0.018 (-0.093,0.006)	< 0.05
	22	Rt DLPFC	0.014 (0.005,0.024)	-0.011 (-0.076,-0.002)	< 0.05
	37	Lt M1	0.015 (0.006,0.068)	0.001 (-0.016,0.016)	< 0.05
	44	Rt M1	0.011 (0.006,0.031)	-0.019 (-0.035,0.003)	< 0.05
CB	15	Lt DLPFC	0.022 (0.006,0.066)	-0.036 (-0.057,0.012)	< 0.05
	32	Lt PMC and SMA	0.014 (-0.004,0.029)	-0.008 (-0.023,0.016)	< 0.05
	35	Rt PMC and SMA	0.019 (-0.007,0.137)	-0.013 (-0.041,0.016)	< 0.05

**Table 6:** Comparison of results of brain activity (Oxy-HB) between the different conditions Ch channels; Lt, left; Rt, right; Oxy-HB, oxy-haemoglobin; ROI, regions of interest; DLPFC, dorsolateral prefrontal cortex; PMC, premotor cortex; M1, primary motor area; SMA, supplementary motor cortex; MI, motor imagery; PP, physical practice; CB, combination between motor imagery and physical practice. P-values: False discovery rate corrected.

Ch	ROI	MI	PP	CB	Comparison	P-value
		Median (Q1, Q3)	Median (Q1, Q3)	Median (Q1, Q3)		
15	Lt DLPFC	-.007 (-.103,0.017)	-0.018 (-0.093,0.006)	-0.036 (-0.057,0.012)	MI vs PP and CB	> 0.05
					PP vs CB	< 0.05
22	Rt DLPFC	-0.008 (-0.028,0.006)	-0.011 (-0.076,-0.002)	-0.007 (-.056,0.021)	MI vs PP	< 0.05
					CB vs MI and PP	> 0.05
32	Lt PMC and SMA	-0.006 (-0.016,0.010)	-.007 (-.023,0.004)	-0.008 (-0.023,0.016)	MI vs CB	< 0.05
					PP vs MI and CB	> 0.05
39, 35	Rt PMC and SMA	-0.003 (-0.017,0.011)	0.003 (-0.016,0.013)	-0.013 (-0.041,0.016)	MI vs CB	< 0.05
					PP vs MI and CB	> 0.05
37	Lt M1	0.001 (-.017,0.013)	0.001 (-0.016,0.016)	-0.013 (-0.050,-.003)	MI vs CB	> 0.05
					PP vs MI and CB	< 0.05
44	Rt M1	0.000 (-.075,0.023)	-0.019 (-0.035,0.003)	-.019 (-.050,0.025)	MI vs CB	> 0.05
					PP vs MI and CB	< 0.05

**Table 7:** Brain activity (deoxy-HB) under all conditions Ch channels; Lt, left; Rt, right; Deoxy-Hb, Deoxy-haemoglobin; ROI, regions of interest; DLPFC, dorsolateral prefrontal cortex; PMC, premotor cortex; M1, primary motor area; SMA, supplementary motor cortex; MI, motor imagery; PP, physical practice; CB, combination between motor imagery and physical practice. P-values: False discovery rate corrected.

Condition	Ch	Anatomical label	Pre	Post	P-value
			Median (Q1, Q3)	Median (Q1, Q3)	
MI	32	Lt PMC and SMA	-0.006 (-0.023,-0.000)	0.003 (-0.004,0.018)	< 0.05
	39	Rt PMC and SMA	-0.020 (-0.051,-0.004)	0.003 (-0.004,0.019)	< 0.05
PP	22	Rt DLPFC	-0.014 (-0.055,-0.010)	0.009 (-0.012,0.070)	< 0.05
CB	32	Lt PMC and SMA	-0.020 (-0.073,0.001)	0.005 (-0.005,0.017)	< 0.05
	35	Rt PMC and SMA	-0.020 (-0.079,0.008)	0.009 (-0.005,0.026)	< 0.05
	42	Lt M1	-0.029 (-0.107,0.002)	0.017 (-0.010,0.058)	< 0.05

## Discussion

This study explored motor learning through three methods (MI, PP, and CB) using an SRTT. It evaluated their impact on physical performance and brain activity, focusing on metrics including premotor time, reaction time, and accuracy rate. This study employed fNIRS to track the changes in oxy-Hb levels pre- and post-training using different methods. Considerable improvements in reaction times were observed after training, with reductions in premotor and reaction times, particularly in the CB condition. In this study, the majority of participants were female. However, a previous study found that although male participants had slightly longer reaction times (RT) than females, the difference was not statistically significant [24, 28]. Brain activity analysis indicated reduced oxy-Hb concentrations, highlighting motor learning across all conditions and the complexity of brain activation during training. These findings underscore the benefits of combining MI with PP to enhance physical performance and motor learning and contribute to a deeper understanding of effective motor learning strategies and their neural bases.

Behavioural parameters

Reaction and Premotor Time

In this study, we observed a significant decrease in premotor and reaction times (RT) following training across all conditions, although there was no notable change in the correctness rate. This outcome aligns with the investigation by Olsson et al., which explored the impact of 72 min of training on a sequential finger-tapping task, evaluating PP, MI, and a combined approach [29].

Although significant improvements were observed, a small but notable advantage was found in the CB condition compared to the PP and MI conditions for RT. Conversely, the PP condition exhibited a small but significant advantage over both the MI and CB conditions for premotor time. However, our study revealed distinct trends when each condition was compared individually. Specifically, the CB condition resulted in the fastest reaction time, whereas the PP condition resulted in the shortest premotor time. In this study, the shortest premotor time (PMT) was observed in the physical practice (PP), motor imagery (MI), and combined (CB) conditions, suggesting enhanced efficiency in central motor planning across all interventions.

In the PP group, repeated movement likely reinforced feedforward control, enabling faster execution with reduced reliance on feedback, an effect supported by activation in regions such as the premotor cortex, supplementary motor area (SMA), and cerebellum [8]. Interestingly, the CB group (75% MI, 25% PP) also demonstrated PMT improvements, implying that MI can facilitate motor planning even when paired with minimal physical practice. Although MI lacks sensory feedback, it recruits similar planning-related cortical circuits and is particularly effective when the imagery is vivid and goal-directed [16]. However, the absence of external feedback during MI may limit its impact on externally cued tasks, which rely on stimulus-response integration. 

Notably, PMT was shorter in MI compared to CB, possibly due to reduced cognitive interference during uninterrupted imagery. The CB condition, which involves alternating between mental and physical practice, may introduce variability in imagery quality and increase cognitive load, slightly attenuating MI’s neural efficiency. These findings highlight the importance of imagery clarity, task-specificity, and consistent application when using MI in motor learning interventions. In a bimanual reaction test requiring coordinated responses, participants demonstrated no discernible differences in hand-related responses despite variations in sequence recognition [13]. In a study conducted by Kraeutner et al. (2016), the efficacy of MI without associated physical practice on reaction time, representing implicit sequence learning, was examined [15]. The study found that MI could enhance motor skill acquisition independently from physical practice. Although physical practice showed a greater decrease in RT after the same training period, the difference was not significant. Therefore, motor imagery has the potential to facilitate the motor learning process, specifically intrinsic sequence learning, similarly to physical practice. 

It is possible that stimulus-response (S-R) mapping played a role in the observed RT reductions; however, implicit sequence learning remains a strong explanation. Prior research has shown that MI can support sequence learning [15], and our findings are in line with this idea. The fact that RT improvements occurred without explicit awareness of the sequence further supports the role of implicit learning mechanisms. This suggests that MI may engage similar underlying processes as other forms of implicit learning, reinforcing its potential as a valuable tool for skill acquisition. Implicit learning, as observed in tasks such as the SRTT, is characterized by improvements in reaction time without the participant's conscious awareness of the stimulus sequence. In contrast, explicit learning occurs when participants are consciously aware of the sequence order [16]. In our study, which focused on implicit learning, no awareness of the stimulus sequences was detected among the participants. Results indicating improvement in motor skills suggest that implicit learning alone may be sufficient to encourage sequence learning tasks [30].

While MI has been found to support sequence learning, its effects are generally weaker compared to physical practice. However, MI also offers unique advantages, such as reduced retroactive interference and greater transfer to novel conditions. Some studies suggest that MI leads to slower initial learning but results in long-term retention and successful skill acquisition. The effectiveness of MI in sequence learning may also depend on the type of imagery used. Kinesthetic MI, where individuals mentally rehearse movement sensations, appears to be more effective in reinforcing motor representations and aiding sequence recall than visual MI, where movements are imagined from an external perspective [21]. However, some research suggests that both approaches may ultimately lead to similar learning outcomes [24].

It is well established that practising MI alone, even without PP, can yield superior results compared to no practice at all [15]. In our study, participants engaged in MI from a first-person or kinaesthetic perspective, resulting in the observed enhancements in reaction times. These findings collectively suggest that kinaesthetic MI activates the sensorimotor network, facilitating fundamental motor skill learning and enhancing reaction time, while potentially mitigating neuromuscular fatigue during high-speed tasks [31]. Kraeutner et al. investigated the efficacy of MI in enhancing motor skill acquisition independent of PP and found comparable improvements in reaction time between MI and PP [15]. Their exploration of practice order also revealed decreased kinematic variability when MI preceded PP. The involvement of internal models elucidates the neural mechanisms underlying the simulated interactions between the motor output and external objects during MI. These models play a vital role in understanding the motor performance improvements following MI. While real execution integrates feedback information from the sensorimotor system with forward internal models for precise movement, MI relies solely on internal models, resulting in a slightly lower precision than actual execution [32, 33].

Integrating MI with PP has emerged as a promising strategy, often yielding superior outcomes compared with employing each method independently. Studies investigating the optimal ratio of MI and PP have posited that a higher proportion of MI, especially in challenging tasks, contributes to greater motor enhancement [34]. Heena et al. scrutinised the influence of varied MI rates on learning tasks involving bilateral hand movements and observed that a heightened rate of MI (75%) extensively bolstered motor learning, particularly in intricate tasks [35]. Additionally, MI complements PP by mitigating physical fatigue during prolonged sessions. However, excessive MI rates may diminish movement performance, indicating potential challenges in sustaining attentional focus throughout the learning trajectory. Consequently, while MI holds promise for fostering motor learning, its fusion with PP necessitates the close consideration of task complexity and individual attentional demands [34, 35]. Therefore, our findings reveal noteworthy enhancement following combined practice, with comparable improvements observed between MI and PP methodologies. Therefore, the combination of MI and PP enhances physical performance by improving RT, as demonstrated in this study.

Our investigation showed a substantial reduction in premotor time across all three post-practice conditions, with the most substantial decrease observed in the PP condition. Premotor time specifically signifies the central nervous system processes and spans the preparatory phase following stimulus presentation, which is influenced by factors such as motivation and task complexity [10]. Kwon et al. [1] documented decreased processing time over three consecutive days of practice through both PP and MI, suggesting modifications in the premotor and motor phases. However, they observed refined differences in RT alterations between the two conditions. Correspondingly, our study also noted a marked reduction in premotor time after training, referred to as processing time in Kwon's investigation, across all conditions, although the fastest reduction was observed in the PP condition.

The development of effective motor skills depends on repetitive practice and sensorimotor feedback to enhance neural plasticity. While PP offers real-time movement feedback, aiding error detection and correction, MI lacks immediate feedback mechanisms. Consequently, PP remains the preferred method for enhancing physical performance, owing to the incorporation of error correction and sensory feedback. During physical training, the inverse model generates neural commands, whereas the forward model predicts the forthcoming arm states and their sensory consequences. This motor prediction, observed in executed and simulated actions, could explain why PP elicits greater improvement than mental practice. Notably, the consistent reproduction of imagined movements is aided by afferent-efferent information stored in working memory, contributing to reduced timing variability in covert movements when overt execution precedes covert rehearsal [15, 36]. In clinical practice, these findings could be valuable for both training and rehabilitation. While physical practice is more effective in reducing premotor time and improving reaction times, MI also plays a crucial role, particularly in enhancing implicit sequence learning. Incorporating MI with PP training regimens could lead to motor performance improvements comparable to those achieved through physical practice alone.

Accuracy rate

In this study, we focused on the critical aspect of movement data accuracy concerning the comprehension of motor learning. Surprisingly, we found no discernible differences among the three conditions even after the training session. These outcomes align with those of earlier research on bimanual sequence reaction time training, which similarly reported no discrepancies in response times between the left and right hands after extensive practice [37, 38]. Despite the marked reductions in the response times observed with both hands, the error rates remained constant. This observation suggests that practice primarily improves response times by refining participants' ability to sequence stimuli rather than enhancing general factors such as task familiarity.

Brain activity

This study reveals a notable finding, described by the reduction in oxy-Hb concentration after training in several brain regions, including the DLPFC, PMC, SMA, and M1. This decrease signified the occurrence of motor learning under each training condition. The observed faster reaction times suggest varying levels of motor activation, suggesting the involvement of additional brain regions in the learning process beyond basic task performance [39].

Interestingly, in the MI condition, we observed a notable decrease in oxy-Hb levels, specifically in the right DLPFC, left and right PMC, and SMA, from pre-training levels to post-training levels and across all conditions. In addition, when examining the deoxy-Hb results, it was evident that both the left and right PMC and the SMA exhibited a marked increase. 

Assessment strategies for motor learning encompass various techniques, such as action observation and MI, both of which engage neural mechanisms similar to mirror neurons. MI induces neural changes in motor preparation at the movement-programming level involving the non-primary motor cortex [40]. These neurons, predominantly located in the frontal and parietal regions, are implicated in MI, execution, and action observation processes [41]. During the initial stages of learning, there is a cognitive demand for task comprehension, resulting in widespread brain activation in the prefrontal cortex and SMA [42]. Cunnington et al. demonstrated that both MI and execution activate the SMA during the premotor phase, as evidenced by electroencephalogram recordings. Notably, participants who underwent PP before engaging in MI exhibited heightened SMA activity during motor preparation, indicating enhanced cortical engagement, even during novel tasks in imagery sessions [43].

The results from the PP condition revealed a considerable decrease in activity, as indicated by reduced oxy-Hb levels, particularly in the left and right DLPFC and the left and right M1, following training across all conditions. In the present study, no significant changes were observed in the PMC, contrasting with previous research that reported increased PMC activation during PP. This discrepancy may be attributed to several factors. One possibility is task familiarity or simplicity; the task was relatively simple or overlearned, participants may have relied more on automated motor programs involving the primary motor cortex and subcortical structures, reducing the need for PMC-driven planning. Additionally, while earlier studies often examined PMC activity during the initial stages of motor learning, when planning demands are higher, this study assessed activation pre- and post-training, potentially overlooking transient PMC engagement that occurs during active skill acquisition. Thus, the absence of significant PMC activation may reflect a shift toward automaticity, task-specific characteristics, or methodological constraints in capturing short-lived planning-related neural activity. Additionally, there was an increase in deoxy-Hb levels in the right DLPFC. In contrast, motor learning hinges on repetitive practice, enhancing performance without requiring verbal articulation, thereby inducing alterations in motor cortical activity [44]. However, the difference in brain activation between physical and mental practice may be influenced by the absence of sensory feedback during imagining. The primary motor cortex plays a pivotal role in controlling fine movements, exhibiting heightened activation during sequential movements compared with ordinary motor tasks. Notably, the decreased response time observed after practice with sequential tasks correlated with heightened activation in this cortex, suggesting its involvement in implicit sequence learning [16]. Mitani et al. investigated brain regions related to executive function, including the DLPFC, parietal lobe, inferior frontal gyrus, and premotor cortex, and found decreased post-learning activity, indicating a discordance between learning progression and brain activity [42]. Typically, in the early stages of training, which may span a few minutes, the average magnitude of neural activation in the M1 decreases [45]. Given that our study's motor training duration was in its early stages, the observed decrease in neural activation in M1 aligns with our expectations.

In the CB condition, marked reductions were observed in the left DLPFC and both hemispheres of the PMC and SMA after training across all conditions. In addition, upon reviewing the deoxy-Hb results, it became apparent that there was a notable increase in both the left and right PMC and SMA, as well as in the left M1, indicating remarkable changes in brain activity. Previous research has shown that learning new motor sequences engages multiple brain regions, such as the prefrontal, premotor, anterior cingulate, and parietal cortices [46]. Specifically, the DLPFC, anterior cingulate cortex, and dorsal premotor cortex play crucial roles in the initial acquisition of an eight-finger sequence, with the DLPFC and right PMC reflecting automated movement execution without the need for attentional control [47]. Earlier investigations reported similar decreases in DLPFC activation during skill acquisition. Furthermore, pre-SMA neuronal activity was notably increased during the early learning phase and gradually diminished as proficiency improved. A comprehensive systematic review and meta-analysis delved into the influence of practice duration on brain activity patterns during motor skill acquisition, classifying studies based on practice duration into short (≤1 h) sessions. Consistently reduced activity has been noted across various practice durations, particularly in the bilateral prefrontal cortex and left presupplementary motor area [39].

Limitation

One limitation of this study is that while the SRTT was chosen to assess motor learning under different training conditions, our primary focus was not on sequence learning specifically. However, the observed reduction in reaction times across conditions suggests that motor learning effects extended beyond simple stimulus-response mapping. Prior research (e.g., Robertson, 2007) has highlighted that both implicit and explicit sequence learning can influence RT, and our findings align with this perspective. That said, without a fully randomized control condition, it remains difficult to completely isolate the effects of sequence learning. Future studies will address this by incorporating a more rigorous control design to better distinguish the contributions of sequence learning from general motor adaptation. Regarding the response rate, we found no significant differences, which aligns with findings from previous research. However, further explanation is needed to clarify these results in future studies.

The crossover study design used in this study may not adequately address baseline characteristics as factors influencing the study results. Some studies suggest that the effects of mental practice may persist for up to a month. The practice intensity in this study was significantly lower compared to previous studies, as it involved only a single day of practice. Moreover, evidence indicates that a seven-day washout period is often sufficient to mitigate carryover effects in similar experimental designs [24]. Thus, the seven-day washout period implemented in this study is considered adequate for the given protocol. 

Although the number of participants in this study reached the target for the sample size calculation, the small sample size may still be considered a limitation. Furthermore, factors such as emotional state, mental fatigue, and personal factors cannot be controlled, even within a homogeneous population. As a result, these factors may disrupt the reaction time.

The analysis of brain activity was limited to select brain regions and did not encompass other areas that may be potentially important for motor imagery and physical practice. To date, a limitation of fNIRS is its inability to detect activity in subcortical regions.

The study opens avenues for further research into the mechanisms underlying motor imagery and physical practice, particularly in different populations, such as older adults, individuals with neurological conditions, or those with varying levels of cognitive function. Future studies could explore how different proportions of MI and PP in training programs affect motor learning outcomes or how these methods influence long-term retention of motor skills.

## Conclusions

In summary, our study observed remarkable improvements in reaction times post-training across all conditions, with notable decreases in premotor and reaction times, but no significant change in the correctness rate. However, when each condition was analysed individually, distinct trends were observed. Specifically, the CB condition showed comparable improvements in enhancing physical performance, as seen in our study, through improved reaction times. By analysing brain activity, we noted reduced oxy-Hb concentrations across various brain regions, indicating motor learning across all conditions. CB resulted in reduced activity in specific brain regions after training, highlighting the complexity of brain activation during motor learning. Overall, our study underscores the potential benefits of integrating MI with PP to enhance physical performance and motor learning, as evidenced by improvements in reaction times and brain activity. These findings contribute to a deeper understanding of effective motor-learning strategies and their neural reinforcement. The findings highlight the role of neuroplasticity in motor learning, showing that repetitive practice, whether physical or mental, can lead to neural adaptations that enhance performance. This supports the idea that cognitive and physical training can be synergistic, promoting more effective learning and memory consolidation.
